# The Hospital. Nursing Mirror

**Published:** 1898-04-16

**Authors:** 


### "The Hospital" Nursing Mirror.

Being the Nursing Section of "The Hospital."

[Contributions for this Section of "The Hospital" should be addressed to the Editor, The Hospital, 28 & 29, Southampton Street, Strand, London, W.C., and should have the word "Nursing" plainly written in left-hand top corner of the envelope.]

### News from the Nursing World.

### THE PRINCESS OF WALES AND THE CHILDREN.

The Princess of Wales has received, in reply to her appeal to children for help towards the Prince of Wales's Hospital Fund, a letter enclosing a subscription of 3s. 9½d. from the members of the cripples' class of the Ragged School Union and Shaftesbury Society, Ratcliff Highway. The Princess has acknowledged it as follows: "Miss Knollys is desired by the Princess of Wales to thank the little cripple children very much for their kind contributions to the Prince of Wales's Hospital Fund, and to assure them that their small offerings are more highly appreciated by their Royal Highnesses than the most costly gifts of those less afflicted than themselves. The Princess is sure that the little cripples will have great satisfaction in feeling that by their own self-denial they are sending help to their fellow-sufferers."

### ST. LUKE'S HOSTEL.

An appeal is being made for funds to enable the Hostel of St. Luke, the nursing home devoted to the use of the clergy and their families, to be moved to new and larger quarters. The home has not been in existence very long, but that it is needed is amply testified to by the number of applications received, many of which have to be refused on account of lack of space. The executive committee have opened a fund for a new home, a sum of £5,000 at least being required. In the words of the Archbishop of Canterbury, "the Hostel of St. Luke is doing excellent work, and deserves the support of all charitable people."

### GOOD NEWS FOR NURSES.

The delightful "Home of Rest" for Nurses at Langleybury, which proved such a boon to many tired nurses last year, has most kindly been reopened by Mr. Loyd. The four vacancies have been placed by Mr. Loyd at the disposal of the London Hospital nurses until the end of April.

### "THE NURSES' JOURNAL."

This little paper is now well established as a monthly publication, and we think those responsible for its production should be congratulated on the excellence of its tone and the interesting matter it contains. *The Nurses' Journal* is just what an Association journal should be, and we feel sure it must be welcomed every month by the nurses of the Royal British Nurses' Association. Miss Lloyd, matron of the Children's Hospital, Birmingham, in "A Plea for No Outdoor Uniform when off Duty," in the number for April, gives expression to opinions with which many will cordially agree. In these days, when active outdoor exercise is so easily within the reach of women, and is acknowledged on all sides to be the best means of securing a healthy tone of mind and body, it is surely well that nurses, whose work must be more or less trying, physically and mentally, should be able to make the best use possible of their off-duty hours. Yet what garb could be more unsuitable for cycling, tennis, or golf than that of a nurse? There is no doubt, too, as Miss Lloyd points out, that the conspicuousness of a nurse's dress brings upon her notice with which she could very well dispense. It is hard for a nurse in her hours of recreation to feel that the public eye is on her, noting all she may do or say, speculating as to her hospital and her work, and so on; besides the fact that the outward proclamation of her calling makes it very difficult for her to steer clear of "shop" when she is amongst her friends. It is very different for private or district nurses when on duty. Here there is everything to be said in favour of a distinctive uniform. But the rule that nurses must wear uniform when off duty is one that is wisely and rapidly being discontinued at most of our hospitals.

### EAST HAM.

The scheme for establishing district nurses, a cottage hospital, and a dispensary at East Ham was discussed at a public meeting the other day, in the course of which some opposition on various points was offered by the medical men of the district, who considered that they were not properly represented on the committee. A sum of money has been collected, and a committee formed, while Colonel Burges has offered a house—the Clock House—and a site. After a good deal of discussion it was finally decided to start the district nursing part of the scheme first, using the Clock House as the home, and to add to it the dispensary and cottage hospital. The committee is to be enlarged.

### HAMPSTEAD GUARDIANS ON NIGHT NURSING.

We are glad to see that at a recent meeting of the Hampstead Board of Guardians a resolution was brought forward "that no nurse, except the night-sister, shall be required to serve on night duty in the sick wards more than one month in three." Mr. Cropper, who was responsible for the motion, quoted Dr. Milson Rhodes, chairman of the Chorlton Union, Manchester, as his authority for the axiom that one month's night nursing in three was sufficient for any nurse to undertake. Mr. Cropper asked the board to vote for his resolution, "as it was merciful to the nurses and considerate to the sick." After some discussion, it was decided to refer the matter to the Nursing Committee for consideration and report.

### DISTRICT NURSING AT HAGGERSTON.

Lady Frederick Cavendish made an eloquent appeal at the eighth annual meeting of the Haggerston and Hoxton District Nursing Association for the money necessary to enable the committee to extend their work, the scope of which has greatly increased of late. In such a crowded part of London there is always room for the employment of more nurses, if the money to maintain them is only forthcoming, and evidence of the value of their helpful presence in the homes of the poor is patent for all who run to read. Help, therefore, can hardly be too liberally meted out to such associations. One nurse has been added quite recently to the staff at Haggerston, but before there can be any further increase it will be necessary to enlarge the home.

### NURSING NECESSARIES FOR CENTRAL AFRICA.

In connection with the Universities' Mission to Central Africa there exists a "Hospital Fund," the object of which is to supply each station in the Mission with drugs, and medical and nursing comforts and appliances of all sorts, without drawing on the general funds for this most necessary purpose. The fund was started in the Mission's early days, and its income for the past ten years has averaged a little over £300 a year; but this sum is now quite inadequate to provide for the needs of the many new stations, with their several hospitals and dispensaries, which have been established in Zanzibar and on the mainland. The greatest medical and nursing skill is powerless in such a deadly climate unless the medicaments which this fund provides are at hand in plenty, and for this its income must be more than doubled. Donations and subscriptions should be sent to Miss S. Phillpotts, Halsey House, Red Lion Square, W.C., the hon. secretary and treasurer of the fund; or to the secretary of the Mission, the Rev. D. Travers, 14, Delahay Street, S.W.

### NURSING IN THE PUNJAUB.

The Punjaub Nursing Association begins its work this week under the management of Miss Macpherson, who has accepted the post of Lady Superintendent. The Up-County Nursing Association is sending out three nurses from England, the first of whom has by this time reached Kasauli, where the home has been established.

### THE JUBILEE NURSES' FUND IN CARNARVONSHIRE.

Carnarvonshire has distinguished itself during the year of the Diamond Jubilee by subscribing a larger sum for the Queen Victoria Jubilee Nurses' Institute than any other county in either England or Wales. The amount reaches £1,706. After paying all expenses, and handing over 30 per cent. to the Jubilee Institute, there remained £1,159, which will be vested in trustees (two gentlemen and three ladies). The interest will be devoted to encouraging and aiding nursing work throughout the county. By this means the object of the fund will be attained, namely to keep fresh and green the memory of the long and glorious reign of our Sovereign.

### LEICESTER INSTITUTION OF TRAINED NURSES.

An influential gathering assembled to hear the report of the Leicester Institution of Trained Nurses. Dr. Carr-Glyn, Bishop of Peterborough, took the chair. For 32 years the society has been working on the same lines, providing private nurses for those who can afford to pay them, and district nurses for the poor. The earnings of the two are kept separate, with the result that whilst the finances of the private nursing staff are satisfactory, the balance to the credit of the district nurses is too small. Earnest appeals were made by several speakers for increased subscriptions to extend this part of the work. The demand for the nurses on both staffs has been constant, and the lady superintendent has had to refuse private cases because she had no one to send to them. The committee intend increasing this staff by adding to it fully-trained nurses holding a three-year qualification. The working of the district nurses has grown in usefulness by their acting more in connection with the medical men of the town. Frequently, with the nurses' assistance and after care, operations are successfully performed in the homes of the patients. This change has stimulated the zeal and interest of the nurses in their work, and has made them still more popular with the people whom they serve. The pressure of work varies considerably, and District VII. is particularly trying, as it lies low and is thickly populated. Unfortunately the nurse-in-charge, who was both popular and efficient, has been compelled to resign, her health having again given way under the strain. During the year the sum of £25, one-half of a legacy by Miss Hirst, has been added to the sick fund, which now amounts to £50; the committee have expressed a wish for further donations to this fund. The other half of Miss Hirst's legacy was for the district nurses, to whose support it has been applied.

### A SWEEPING REFORM.

If in some of the Irish workhouses the Guardians exercise their ingenuity in order to evade the new order of the Local Government Board others loyally endeavour to fulfil its requirements. Tullamore is of the latter kind. At a recent meeting it was resolved to advertise for a nurse with midwifery qualifications at £20 a year, and 11 attendants at a salary of £10 each per annum. Wages in Ireland are low, and £10 a year ought to command very good rough material. Therefore, if the Guardians are lucky in their trained nurse, for whom, by the bye, they ought to offer more, Tullamore will soon be fairly well nursed.

### SHORT ITEMS.

The St. Asaph Board of Guardians, in response to a request for a grant from the hon. secretary of the Abergele District Nurse Fund, have agreed to subscribe two guineas to the fund.—A District Nursing Association is about to be started at Stradbroke, and will include the parishes of Wilby and Horham.—The Larne District Nursing Society has just held its ninth annual meeting, at which a very satisfactory report was presented.—The Lord Provost of Edinburgh and the members of the Committee of Public Health gave an "At Home" recently to the representatives of the nursing staffs in all the city hospitals.—Subscriptions and donations to the Kilmarnock Nursing Association show an increase over those of last year, as do also the cases nursed and the visits paid. A fourth nurse has been added to the staff.—A Sick Nursing Association is being started at Dalton.—A provisional committee has been formed at Cleator with the object of providing district nurses for the neighbourhood. It is proposed to begin with three nurses, and to obtain for the scheme, if possible, the support of the local mine owners, clubs, etc.—Nurse Sinclair, a Queen's nurse, has been appointed district nurse at Banchory, N.B, Nurse Ellen Garden, whose temporary services during the winter have been very much appreciated, returning to her work at the Children's Hospital, Aberdeen.—A concert in aid of the Westminster Hospital will be given by the Westminster Orchestral Society, in conjunction with the Streatham and Reigate Choral Societies, under the patronage of the Duke of York.

### Lectures to Surgical Nurses.

By H. A. LATIMER, M.D. (Dunelm), M.R.C.S. of Eng., L.S.A. of London, Consulting Surgeon, Swansea Hospital; President of the Swansea Medical Society; Lecturer and Examiner of the St. John Ambulance Association, &c.

### XXIX.—OVARIAN TUMOURS: THEIR NATURE AND RESULTS—OVARIOTOMY: PREPARATION FOR AND SUBSEQUENT TREATMENT BY NURSE.

I SHALL pass on at once to tell you something about ovarian tumours, and shall speak of that great operation of ovariotomy, which was at one time so rarely performed, but which has now become a constant procedure in surgery. The ovaries are two small oval-shaped bodies which lie in the pelvis, one on each side of the womb, with which they are connected by ligaments and channels of communication called the Fallopian tubes. Their function is to form ova or eggs which are discharged at the monthly periods; the ova are conveyed through the Fallopian tubes into the uterus, where they should remain and develop into children if they have become impregnated. This is the normal process which, like all other processes in the body, is subject to deviations from the safe course. There is no call to deal with these deviations, however, as my theme now is the diseases which take place in the ovaries themselves, and the operations which are undertaken for their cure.

The ovaries are peculiarly liable to change in structure, and to become converted into cysts or fibrous tumours. I would like to give you a definition of a tumour before I go any further. The word really means a swelling; but every swelling is not a tumour. When pathologists call a swelling by that name they mean one which has a growth of its own, independently of the part in which it lies. Thus, if tumours form in the ovaries, they grow larger and larger as time goes on, and they lead a life of their own, and are quite independent of other organs around them; they behave, in short, like selfish people do in the world, for they elbow aside and push ush out of their way everything in their neighbourhood. They grow with remarkable rapidity, and before long rise up into the abdomen, and, distending its walls, make their presence known by a general protuberance of that region of the body. Growing in this way they exhaust the patient by interfering with the functions of other organs, by pressing upon them, and by the attacks of inflammation which they excite in the peritoneum, or elsewhere, in the neighbourhood; and they make such great calls upon the system at large for their support and increase that they lead to a general loss of strength. As they are irremovable by any medicinal treatment they have to be dealt with by operations. If they are cystic they contain fluid; for cysts are bladders filled with more or less thick fluid secreted by their walls. Formerly it was customary to tap these cysts from time to time and to empty them of their contents, but as tapping is only a palliative measure and one which in no way cures, and as the operation of ovariotomy, in which the tumours themselves are removed, frees the patient from her disease, the practice has been discontinued. I have, in the old days, seen a patient suffering from cystic disease of the ovaries tapped as many as 24 times in twelve months, with a total removal of 38 gallons of fluid; the little operation had to be repeated at shorter and shorter intervals until she sank, worn out with the continual demands made upon her strength by this disease. When it was possible under better methods and modes of operating to deal with these cases by free incision of the abdominal wall and peritoneum, and removal of the tumours, tapping was discontinued. I need not give any details of the steps taken in the operation as they do not fall within your province, but I must tell you what you will have to do in the wards, before and after the operation, and in the operating-room, while the patient is on the table. You will prepare her by attending scrupulously to her personal cleanliness, especially with antiseptics at the abdominal walls, and by clearing out the bowels on the day of the operation, or on the evening preceding it if the operation is to be performed very early in the morning, with an enema; and she should be spoken to cheerfully and reassuringly as to the success and cure which are likely to follow what will be done to her. Not later than four hours before the operation she should have her last supply of nourishment, consisting of a cup of beef tea, or some other such lightly digestible and easily absorbable food. She should be warmly clad in her night-dress, covered by a flannel dressing-gown, and woollen stockings, and when she lies down on the operating table the former should be rolled up and kept in place by safety-pins, over which the mackintosh sheeting, which is used to keep her clothing from being soiled and wetted during the subsequent proceedings, should be attached. Of late, a waterproof sheeting has been devised, which has a bag-like arrangement at each side of the operating table terminating in two tubes; the lotions and discharges are at once conducted away from the patient by these drains. Your great care will be on account of the sponges which will be used during the operation. You should carefully count these before they are to be used, and again when the operation is completed, to be sure that the same number are then in your charge; a neglect of this precaution has, on one or two occasions, resulted in a sponge being left behind within the abdomen. This mischance has happened in this way. It is customary, in order that all blood and discharges shall be absorbed from the surfaces of the exposed intestines, and from the depressions within the pelvis, to leave some sponges within the abdomen whilst inserting the final stitches in the abdominal walls; just before these are drawn tight and the wound is secured, the sponges are withdrawn. Now the operator turns to you and asks, "Are the sponges all right, sister?" and you count them and make sure that the original number under your charge remain there. During the operation these sponges must be squeezed out of an antiseptic lotion which is of the warmth of 99 degrees, and they should be handed in as dry a state as possible to the surgeons, so that they may possess their full powers of absorption; and all sterilised water or lotions which are to be used for irrigating the abdomen must be of a similar warmth.

On the removal of the patient to her bed she is to be placed on her back, with her knees raised and supported by pillows, and her head and shoulders also raised. Hot-water bags or bottles are to be placed near her calves and feet, and her body is to be protected from the pressure of the upper bedclothes by a large cradle. Her temperature is to be taken and charted every four hours. Unless shock is very severe, and you have received some special directions about rectal or other feeding, you are to give nourishments in the way I have described in my remarks on that subject after operations for strangulated hernia. The catheter must be passed by you every eight hours for the first few days, and you must be scrupulously careful to keep this instrument clean, for that purpose rinsing it in an antiseptic lotion before and after use; for, if you neglect to adopt these precautions, you will carry germs of disease into the patient's bladder, and will there set up an attack of vesical catarrh. The patient is not to move in bed without your help, but you may make a little change of her position from time to time if she desires this very much. But, you understand! the change is to be made by you, she lying inertly in your hands. Sickness is apt to give you trouble after the operation, but it generally passes off when the influence of the chloroform has worn away.

### Nursing in Paris Hospitals.

### A.—LAY NURSES.

### IX.—THE BUDGET FOR THE NURSING SERVICE.

IN no country, so far as I know, are such minute and exact details given in the reports of the public service as in France. The ingenuity exercised by the French official brain in taking a batch of figures and twisting them into all sorts of problematical combinations is something marvellous. In spite of this characteristic, all the recent reports of the Assistance Publique entirely ignore the most interesting detail of the nursing system in the hospitals, especially in view of the displacement of the sisters, and that is the respective numbers of male and female nurses. Male nurses are such a small fraction in London hospitals that English critics, in discussing the Paris service, are naturally apt to fall into the error of thinking the subject of lay nurses in France is entirely a woman's question. That this is not the case, the following table of the sexes in each grade of nurses at the various Paris hospitals is of interest:—

| HOSPITALS. | Beds. | Superintendent Nurses. | | | Assistant Superintendents. | | | Assistants. | | | Staff Nurses. | | | Assistant Nurses, Housemaids, and Porters. | | | Hospital Totals. | | |
|---|---|---|---|---|---|---|---|---|---|---|---|---|---|---|---|---|---|---|---|
| | | Men. | Women. | Total. | Men. | Women. | Total. | Men. | Women. | Total. | Men. | Women. | Total. | Men. | Women. | Total. | Men. | Women. | Total. |
| Hôtel Dieu* ... ... | 827 | 4 | 1 | 5 | 1 | 1 | 2 | 5 | 2 | 7 | 6 | 12 | 18 | 89 | 69 | 158 | 105 | 85 | 190 |
| Pitié ... ... ... | 717 | 3 | 7 | 10 | 2 | 11 | 13 | 3 | 8 | 11 | 4 | 9 | 13 | 49 | 51 | 100 | 61 | 86 | 147 |
| Charité ... ... ... | 616 | — | 5 | 5 | 4 | 12 | 16 | 3 | 13 | 16 | 3 | 7 | 10 | 61 | 36 | 97 | 71 | 73 | 144 |
| St. Antoine ... ... | 868 | 3 | 9 | 12 | 2 | 15 | 17 | 2 | 17 | 19 | 6 | 11 | 17 | 71 | 53 | 124 | 84 | 105 | 189 |
| Necker ... ... ... | 475 | 1 | 10 | 11 | 3 | 8 | 11 | 2 | 8 | 10 | 3 | 8 | 11 | 55 | 24 | 79 | 64 | 58 | 122 |
| Cochin ... ... ... | 502 | — | 6 | 6 | 5 | 9 | 14 | 1 | 13 | 14 | — | 11 | 11 | 26 | 55 | 81 | 32 | 94 | 126 |
| Beaujon ... ... ... | 563 | 1 | 8 | 9 | 4 | 14 | 18 | 2 | 9 | 11 | 4 | 10 | 14 | 52 | 48 | 100 | 63 | 89 | 152 |
| Lariboisière ... ... | 905 | 4 | 9 | 13 | 2 | 28 | 30 | 5 | 8 | 13 | 6 | 23 | 29 | 67 | 63 | 130 | 84 | 131 | 215 |
| Tenon ... ... ... | 888 | 4 | 9 | 13 | 1 | 18 | 19 | 3 | 14 | 17 | 1 | 15 | 16 | 41 | 85 | 129 | 50 | 144 | 194 |
| Laennec ... ... ... | 628 | — | 9 | 9 | 3 | 14 | 17 | 2 | 8 | 10 | 5 | 8 | 13 | 38 | 45 | 83 | 48 | 84 | 132 |
| Bichat ... ... ... | 191 | — | 5 | 5 | 4 | 6 | 10 | — | 5 | 5 | 4 | 2 | 6 | 13 | 23 | 36 | 21 | 41 | 62 |
| Andral ... ... ... | 109 | — | — | — | 3 | 2 | 5 | — | 5 | 5 | 2 | 2 | 4 | 5 | 10 | 15 | 10 | 19 | 29 |
| Broussais ... ... ... | 264 | 1 | 2 | 3 | 3 | 5 | 8 | 1 | 10 | 11 | — | — | — | 23 | 24 | 47 | 28 | 41 | 69 |
| Hérold ... ... ... | 100 | — | — | — | — | 1 | 1 | 1 | 4 | 5 | 1 | 5 | 6 | 8 | 9 | 17 | 10 | 19 | 29 |
| St. Louis* ... ... ... | 1000 | 5 | 2 | 7 | 5 | — | 5 | 4 | 3 | 7 | 9 | 14 | 23 | 110 | 93 | 203 | 133 | 112 | 245 |
| Ricord ... ... ... | 317 | 4 | 1 | 5 | 3 | 1 | 4 | — | — | — | — | — | — | 27 | 1 | 28 | 34 | 3 | 37 |
| Broca ... ... ... | 294 | — | 5 | 5 | 3 | 7 | 10 | 1 | 7 | 8 | 3 | 4 | 7 | 4 | 27 | 31 | 11 | 50 | 61 |
| Maternité ... ... ... | 413 | 1 | 7 | 8 | 3 | 4 | 7 | — | 5 | 5 | 1 | 7 | 8 | 13 | 37 | 50 | 18 | 60 | 78 |
| Baudelocque ... ... | 180 | — | 1 | 1 | 1 | 3 | 4 | — | 3 | 3 | — | 3 | 3 | 6 | 25 | 31 | 7 | 35 | 42 |
| Clinique ... ... ... | 198 | 1 | 2 | 3 | 2 | 2 | 4 | 1 | 2 | 3 | — | 2 | 2 | 6 | 17 | 23 | 10 | 25 | 35 |
| Aubervilliers ... ... | 260 | — | — | — | 1 | 2 | 3 | — | 5 | 5 | 1 | — | 1 | 18 | 13 | 31 | 20 | 20 | 40 |
| Enfants Malades ... ... | 635 | 1 | 8 | 9 | 4 | 18 | 22 | 2 | 11 | 13 | 1 | 12 | 13 | 10 | 97 | 107 | 18 | 146 | 164 |
| Forges ... ... ... | 224 | — | 1 | 1 | — | 2 | 2 | — | 7 | 7 | — | 5 | 5 | 2 | 15 | 17 | 2 | 30 | 32 |
| Trousseau ... ... ... | 602 | 1 | 4 | 5 | 5 | 11 | 16 | 1 | 16 | 17 | 3 | 16 | 19 | 16 | 77 | 93 | 26 | 124 | 150 |
| Roche Guyon† ... ... | 118 | — | — | — | — | — | — | — | 1 | 1 | — | 1 | 1 | — | 2 | 2 | — | 4 | 4 |
| Berck ... ... ... | 750 | — | 2 | 2 | 2 | 10 | 12 | 1 | 14 | 15 | — | 30 | 30 | — | 16 | 16 | 3 | 72 | 75 |
| Maison de Santé ... ... | 310 | 2 | 4 | 6 | 1 | 4 | 5 | 2 | 10 | 12 | 2 | 49 | 51 | 32 | 21 | 53 | 39 | 88 | 127 |
| Totals for the 27 Hospitals | 12954 | 36 | 117 | 153 | 67 | 208 | 275 | 42 | 208 | 250 | 65 | 266 | 331 | 842 | 1039 | 1881 | 1052 | 1838 | 2890 |

* Besides 32 Sisters. † Besides 7 Sisters.

The figures I have obtained myself by personal inquiry, since no printed report, so far as I am aware, has ever included this information. The latest available figures for any expenses incurred for wages of the nursing service of these 25 hospitals are those for 1895, when the total is given as 1,567,978·87 francs. This includes, however, a few other persons besides these nurses proper, such as cooks, carters, &c. It does not include, however, the expense of food, lodging, and uniforms furnished to nurses inside, though it does the allowance for those living outside and all pensions. The same also includes two small hospitals since abolished, or replaced, but of little moment. Taking the figure as it stands, we have a daily average of 33 centimes per bed.

The administration anticipates a serious increase in the expenditure in carrying out the new rules. The Municipal Council may vote an increase of salaries and pensions, but it is not inclined to increase the appropriation. Thus, to meet the deficit, the Assistance Publique is obliged to sell some of its invested funds. Of course, if this goes on indefinitely, bankruptcy will ensue, and the taxpayers of Paris will have to pay the piper.

The amelioration in the treatment of the nursing staff occasioned by the tuberculosis investigation was estimated to entail an additional cost of about 1,400,000 francs. Of this 575,911 francs was for increase of wages; 300,000 francs for reducing the maximum pensions limit from 30 to 25 years of service; and, finally, 508,860 francs for allowing the lower grade nurses to be served at the first-class kitchens. This last item was estimated to entail an additional 50 centimes a day for each of the 2,827 assistant nurses, raising the estimate for their food to 1,384,000 francs. The total pay roll for the nursing staff for 1897 was voted at 2,706,716 francs—a portentous increase to be noted.

No one has been hardy enough yet to estimate the cost of renewing the lodgments in all the hospitals, but it may be set at many millions of francs. It is even impossible to get any estimate of the cost of the installations already made. Such information as I can get, as also certain other new features of the lay nursing budget, I will, however, set forth later when I deal with the question of the comparative cost of the new lay system and the cost under the old *régime* of the Sisters.

EDMUND R. SPEARMAN.

### A Book and its Story.

### SIR JAMES RANALD MARTIN, C.B., F.R.S.

A QUARTER of a century has elapsed since Sir Ranald Martin* died, but his memory still lives with us in connection with the advancement of sanitary reform, a work to which he devoted a lifetime. His memoirs, compiled by his friend Sir Joseph Fayrer, are interesting as the testimony of one whose services in the same cause are pre-eminent.

Sir Ranald Martin was born in 1796 at Kilmuir, in the Isle of Skye—a little island which within the 40 years preceding 1852 had given to the public service 21 lieut.-generals and major-generals, 45 lieut.-colonels, 600 majors, captains, and subalterns, and 10,000 foot soldiers.

James Ranald Martin was destined to follow the military calling, but certain family considerations led him to enter upon a medical career, and in 1813 young Martin proceeded to London, where he entered St. George's Hospital. Here, under Everard Home and Benjamin Brodie, he applied himself assiduously to laying the foundations of an after-career of much distinction. His hospital work over, Ranald Martin became a member of the Royal College of Surgeons, and obtained, through the interests of his uncle, Sir John Macdonald, a commission as surgeon in the Honourable East India Company's service. Quickly following on his arrival in Calcutta, he was appointed to duty at the General Hospital for Europeans, where many of the I.M.S. laid the foundations of the reputations afterwards obtained. The conduct of the young surgeon, from the commencement of his career, was characterised by unflinching devotion to his duties. In recognition of his services, he was speedily appointed to the medical charge of the Governor General's bodyguard, a post of considerable distinction and emolument.

It was even at this early date that young Martin evinced a sense of the importance of hygienic measures by recommending extensive sanitary improvements in the cantonment of his own regiment. His appointment as medical adviser to the bodyguard was made at a period in the history of India when Sir Archibald Campbell, with the military and naval forces under his command, had achieved many successes over the Burmese, under Mande Bandoola. The expedition was rife with tragedy to the troops engaged. Ranald Martin was one of a few survivors at the end of the first year's campaign; 3 per cent. of the British soldiers were killed in warfare, 45 per cent. perished by disease. This campaign proved to be replete with hardships and privations, but, throughout, Mr. Martin conducted himself with his wonted zeal and devotion to duty. He continued to serve with the bodyguard throughout this and the following campaign. In his notes on the Medical Topography of Calcutta, published in 1837, in alluding to the causes of the suffering and loss of health in this expedition, Mr. Martin writes: "It was owing to the ignorance or neglect of military topography that every ultimate object in sending a force to Arakan failed; and it was a similar neglect of medical topography that caused the destruction of that force."

In 1826, Mr. Martin arrived in Calcutta in enfeebled health; but on recovery was appointed, for the third time, assistant surgeon at the General Hospital. Later, he was appointed to the office of officiating surgeon to the Governor-General; thus, after ten years' service, and whilst still a young man, he was elected to hold an important public position. The following year he was appointed examiner at the Calcutta Medical College, and also member of a committee to inquire into subjects connected with the Materia Medica of British India. About this time Mr. Martin presented the Government with a detailed report upon the medical topography, climate, and diseases of Calcutta, Following on this, the appointment of presidency surgeon was offered him, a post which involved alike the duties of a general practitioner and of a consulting physician. Mr. Martin's career in India terminated in 1839, when he returned to England to continue a no less energetic and useful life than he had passed in India. In 1842, he severed his connection with the Indian Medical Service: the following year he was elected a Fellow of the Royal College of Surgeons, and the same year, in consequence of his services in the cause of hygiene in India, he was appointed a member of the Royal Commission of Inquiry into the sanitary condition of large towns and populous districts in England and Wales. To Mr. Ranald Martin is due the proposal that a medical officer of health should be appointed in all our large cities, a measure which, since it has been carried into effect, has proved of much importance as a great step in modern civilisation.

In 1845, Mr. Martin was elected a Fellow of the Royal Society, and in 1848 "he again appears on the scene of public duty, for he was appointed by Government first member of a board to report on the capabilities of the metropolitan workhouses for the reception and treatment of cholera cases. The report of this Commission was printed by order of Her Majesty." Besides active work in the cause of sanitation in the country, Mr. Martin contributed articles to the *Lancet*, amongst which were a series of essays on the diseases of Europeans on their return from tropical climates. But the article which attracted the most attention was a statement of the claims of the medical officers of the army and navy to military rewards and distinctions. It is not possible in the prescribed limits of space at our command to enlarge upon the various works to which Mr. Martin devoted his energies, and which have been so admirably described in the volume now before us, and for these and other interesting mention of Mr. Martin's career we must refer the reader to Sir Joseph Fayrer's account. In 1860 knighthood and the distinction of the third-class of the Civil Order of the Bath were conferred upon the distinguished sanitary reformer. But, as his biographer remarks: "The third class of the Order of the Bath, with simple knighthood, was generally regarded as an inadequate recognition of the services that he had rendered throughout a long lifetime. It is said that he had been recommended by the highest authorities for the second class of the Order, but for some mysterious and unexplained reason, it was refused," and further, Sir Joseph Fayrer continues, "his claims upon the respect of the medical profession and the army are in no degree dependent upon such distinctions, nor could any honours have enhanced the esteem in which he was held by all who knew his merits and his great public services, however much they might regret the unappreciative spirit that withheld them."

During the ten years of life that still remained to him he continued to perform the duties of President of the Medical Board, Physician to the Secretary of State for India in Council, member of the Army Sanitary Committee, and of the Senate of the Army Medical School at Netley, in the formation of which school, as well as in the establishment of the great hospital at Calcutta, Sir Ranald Martin had taken an initiatory part. After his death in 1875 a number of friends and admirers assembled to consider the question of a memorial, the result of which movement was the endowment of a prize known as the Martin Memorial Gold Medal, presented to the surgeon on probation who takes the highest place in military medicine at the competition at Netley at the close of each session, and its presentation has generally been accompanied by some words commemorative of the distinguished physician whose name it bears.

* "Sir James Ranald Martin, C.B., F.R.S." By Sir Joseph Fayrer, Bart. (A. D. Innes and Co., London, 1897.)

### Carnival in a Children's Hospital.

THE idea of keeping carnival in hospital seems strange to English minds, but most natural to Italian ones. The elderly nuns here speak almost regretfully of olden times, when the *infermiere - inserviente* (servant - nurses) acted comedies so well that they gained over 100 frs. by inviting the doctors and their friends, charging them 20 cents a seat. Now such gaieties are not permitted in the large hospitals, only in our small children's hospital a certain amount of observance is still allowed.

There were extra cleanings for some days before *Giovedi grasso*, walls, floors, and furniture being disinfected to please the professors, and then polished to please the nurses, whilst on the vigil the Sister Superior ordered a general bathing of all except the dying. Happily, the wards were fairly light (many children having gone home for carnival), and everyone set to work with a will. Still it was no easy matter to bathe some forty children in a hospital where the bath-room is on the ground floor, and the wards on the first. In summer it is easy to carry the children down to the six little marble baths; but, though the supply of hot-water is abundant, it is not desirable in winter to carry sick children through corridors and staircase of different temperature, from bed to bath. Consequently, only the convalescents were taken to the real bath-room. The majority were washed in a big tub in the little linen room of the wards; but as the water had to be fetched from down stairs, they changed it at intervals whose duration would have scandalised an English nurse. Those little patients too ill to be put in the tub had bed-baths given them by the student nurses. These latter are young Italian girls of the educated classes, who are now beginning, in several of the larger Italian towns, to train for private nursing very much on English lines. The children are very devoted to them, and look on a bed-bath as a great distinction; unlike the "tub," over which so many tears were shed, that to console them the *infermiere* had to promise to come and show themselves at night, masked. This promise elicited shrieks of delight from the elder children, who had been trying to look out of window all day, to see "le maschere." But by seven o'clock all the little eyes were closed, and the servants kept their carnival downstairs, in their own refectory.

This room they had decorated with every ornament on which they could lay hands, beguiling the Sister Superior to lend all the tinsel ornaments put by from the Christmas Tree, nailing up every picture (mainly English) discarded by the children, borrowing the few plants allowed in the wards, and, finally, draping the doorway with curtains of the starched muslin used for bandages, and a strip of red material borrowed from the nun's little oratory.

After supper, the young house doctor and surgeon brought guitar and mandolin, and played to them, whilst those who knew how, danced. Several wore masks, and more or less absurd costumes, masculine as well as feminine, borrowed from brother or cousin, or from the student nurses' *direttrice*. The Hospital Superintendent (a "Chief") had sent cakes and marsala, so they spent the evening as much to their satisfaction as was possible in these degenerate days.

The night sister smiled on them charitably as she passed from ward to ward, remaining alternately in each, to help the *infermiera* in charge, or even sending her to join in the revelry. Italian nuns have the carnival feeling in their veins, and sympathise with it avowedly. "It is carnival, and one can do anything," is still a national sentiment.

The next morning the children were very disappointed at not having seen "le maschere," but were soon consoled by hearing they should have a little carnival themselves. A few ladies had been invited to come and bring them cakes and masks in the afternoon, and the little ones talked of nothing else. Several of the bigger children manufactured masks and dominoes out of gauze or bandages, and great was the excitement when three o'clock brought the visitors with a fine variety of real masks and cakes. All the convalescents crowded round the table, till each eager little face was covered with either a mask with whiskers, or a monkey mask, or a dog mask, or else a black mask, or a mask which was only a huge nose—when they trotted off to their friends in bed, frightening one or two of the more timid till they cried, and had to be comforted with a mask for themselves.

The next important matter was, of course, the distribution of the "le chicche." Such hygienic delights as sponge fingers, chocolate cakes, and simple biscuits had been chosen, so that almost every baby was made happy without risk of indigestion. Only two, in fact, were unable to eat—one, a small girl of six, burnt, so that face and hands were as effectually masked as any carnival revel. No one knows how Pia got burnt; she was sent on here three days ago from the main hospital, where her parents had brought her and left her. They have never been to inquire for her, and, judging from the poor child's language, they must belong to the lowest strata of society. The first day she could only cry for water, and that almost inarticulately, but the second she began to make herself understood, and the third apostrophised the surgeon, sister, and infermiera (whilst being dressed with salicylic acid and vaseline), repeating persistently "Accidente a boi," equivalent to the English "Damn you."

The other small victim was just out of chloroform after amputation of the right foot, but lay quietly watching his friends without begging for either biscuit or mask.

One of the servants next brought in an organetto, a Christmas gift from the six month old baby of the superintendent to her father's poor little patients. The convalescents hastened in from the other wards, some being wheeled (an English rolling chair), others carried, and set upon beds, but all so merry. A waltz was first played, but they, one and all, declined to dance. It is curious how, in some ways, Italians have stronger feeling than the English about not attempting what they cannot do really well. It is a known fact that Italians do not attempt to sing unless they have an exceptional voice and ear, unlike most English maids and men, who are always more or less willing to warble drawing-room songs independent of voice or ear. But it is strange to find the same instinct in these poor children; they declined to dance, saying, "We do not know how." But on changing the waltz to a tarantella, Bice, aged eight, minus a foot, threw down her crutches, and began the wildest gyrations, sometimes catching hold of the bed-rails, sometimes spinning round without any assistance, sometimes whirling herself on her knees, but always in perfect time to the music. Her black hair flew out, and her eyes sparkled; whilst her companions greeted the performance with shouts of delight, even the burnt child sitting up in bed and twisting her poor head so that the one open eye could look out of the slit left amidst the bandages.

One little boy found courage to attempt to imitate *la Bice*; he, too, moved with most perfect rhythm, and on the music changing back to a waltz he let himself be taught the waltz by a visitor, again moving instinctively in time to the measure.

The young house surgeon passed and applauded, also the sister-in-charge, the whole "atmosphere" being as cheerful and home-like as in any English hospital. But, after all, it was Italy and Carnival, as was proved by the rushing in of two strange masked figures, who, as they danced round the ward, were recognised by the children as their *infermiere inserviente*, Bianca and Isolina; whilst a further reminder was given by the doctor being called to attend to an accident case—a young girl accompanied by several youths, and whose forehead was rather badly gashed. This, too, was "fruit of the carnival," the throwing of other and harder things than *corriandoli* being permitted on this, its last day.

### Responsibility for Injury Sustained on Duty.

A case tried recently at the Birmingham Assizes, in which a nurse obtained damages for injuries inflicted on her by a patient, is of considerable interest, seeing that it was urged by counsel, among other pleas, that the plaintiff undertook her employment as nurse to the defendant with full knowledge of her state of mind, and therefore accepted the duties and risks incident to the nursing. It appeared that the plaintiff in May last year was in the employ of a doctor at Worcester as second nurse, her duty being to assist in looking after a Mrs. Green, who had been placed under restraint in the doctor's house. One night Mrs. Green got out of bed and said she was going to walk about the room. The plaintiff tried to reason with her, and the patient then struck her a violent blow on the left eye. In consequence of the injury the plaintiff had to attend an eye hospital in Birmingham, where it was discovered that the retina had been detached and that the vision had been entirely destroyed. The doctor at whose house the affair took place stated that at the time it occurred Mrs. Green was a furious lunatic, dangerous to others, and supposed to have suicidal tendencies. She smashed windows, and threw everything about that came in her way. The jury returned a verdict in favour of the plaintiff for £78. This sum was very much less than what had been claimed, but it is very satisfactory to find that the plea that this was one of the ordinary risks common to her employment as a nurse, and, as the saying goes, "all in the day's work," was not allowed to stand. Many cases occur in which nurses are seriously injured by their patients, and the friends of the latter are only too ready to look upon such risks as included in the bill.

It is all the more satisfactory to note that in this case the assault was committed while the patient was in such a condition of mind that she could not appreciate the nature of her act, for it is just in such circumstances, and in cases of delirium, that nurses run the greatest risks.

### Appointments.

Poplar and Stepney Sick Asylum.—Miss S. E. Polden has been appointed Second Assistant Matron at this institution. She was trained at St. Bartholomew's Hospital, afterwards holding the position of staff nurse there; she then acted temporarily as assistant matron at the Hospital and Convalescent Home, Parkwood, and since January has held the post of night superintendent at the Poplar and Stepney Sick Asylum.

Bolton Infirmary and Dispensary.—Miss Marion Lloyd has been appointed Matron to this institution. Miss Lloyd has latterly been matron at the Cancer Hospital at Manchester, and previous to holding this post was night superintendent and deputy matron at the Royal Albert Edward Infirmary. Miss Lloyd was trained at the Bristol General Hospital.

Kingsbridge (Devon) Cottage Hospital.—Miss C. Lloyd has been appointed Nurse-Matron. She was trained at Newcastle Infirmary, and also in Wellington, N.Z. Since her return from New Zealand, she has been working as district nurse.

### Presentations.

On Miss Anstey resigning the post of matron of the Bolton Infirmary, owing to failing health, she was presented with a very handsome travelling clock by the nurses, and a beautifully-fitted travelling bag by the servants, as well as with many other useful gifts.

### Everybody's Opinion.

[Correspondence on all subjects is invited, but we cannot in any way be responsible for the opinions expressed by our correspondents. No communication can be entertained if the name and address of the correspondent is not given, as a guarantee of good faith but not necessarily for publication, or unless one side of the paper only is written on.]

### SEATS.

"Alureda Follows" writes: I have just read with much interest your article on the much-vexed question of the standing of shop assistants with its bearing towards the standing of hospital nurses. At a large meeting held last week in Manchester, Dr. Sinclair plainly pointed out that the danger to health lay in the monotony of their position. The limbs bear the whole weight of the body, which has no exercise to promote necessary circulation. Nurses are exempt from this; their exercise is varied; and when this is so, being on their feet, more or less, all day is not injurious when the health is fairly good. Nurses suffer more from the effects of hospital air, which induces a tired feeling all over the body, including the limbs. They often mistake the cause of the fatigue. Certainly no nurse could take a lounging position in a ward, and such a position is not necessary when their work and exercise is so varied.

### A BIT OF BUTTER.

"A Matron" writes: With reference to the article on "A Bit of Butter" in the "Nursing Mirror" of last week, may I take the liberty of saying that I have found, after a good many years' practical experience, that the truest economy in hospital administration is to always supply good food of every kind and an ample allowance for every one, care being taken that no waste is allowed. I am quite certain that nurses require good food, served nicely, and in abundance. There will, perhaps, be more (butter, &c.), used, but the health of the individual will be maintained, and in consequence the nurse's drug account will be decreased, to say nothing of the other expenses consequent on illness, her work being at the same time more efficiently performed. In this hospital we do not have allowances. As much butter as required is placed on the table for breakfast, luncheon, tea, and supper; the tables are decorated with plants and fresh-cut flowers (except in the very depth of winter, and even then trails of ivy, the first celandines, primroses, catkins, &c., will during a bicycle ride be eagerly sought out and carried home by the nurses and probationers to the night superintendent, who kindly undertakes the table decorations). Some will, no doubt, wonder where our supply of plants for the dining-room comes from, and perhaps it may be a suggestion to others to tell them that in our "linen room" we have a "rag bag," into which all cuttings and rags go. These are sold occasionally, and the proceeds of the rags provide the plants—which are bought and cared for by our kind, good housekeeper—for the nurses' dining-room, kind friends and nurses sometimes adding a plant or two.

### Minor Appointments.

City of London Union Infirmary, Bow.—Miss Esther Hind has been appointed Superintendent Nurse at this infirmary. She was trained at the Royal Infirmary, Edinburgh, and at the Children's Hospital, Pendlebury. She has been staff nurse at King's College Hospital, sister at the Hospital for Women, Soho Square, matron of the Hulme District Nurses' Home, and matron of the Tunbridge Wells Homœopathic Hospital.

Poplar and Stepney Sick Asylum.—Miss Annie M. Murray, of the General Hospital, Birmingham, has been appointed Night Superintendent (female side) at this institution. Miss Murray has been sister in the Army Nursing Service for the past seven years.

### For Reading to the Sick.

"COME unto Me, all ye that labour and are heavy laden, and I will give you rest."—S. MATTHEW xi. 28.

**Verses.**

All ye who seek a comfort sure
In trouble and distress,
Whatever sorrow vex the mind
Or guilt the soul oppress.

Jesus, who gave Himself for you,
Upon the Cross to die,
Opens to you His Sacred Heart;
Oh, to that Heart draw nigh.

Ye hear how kindly He invites;
Ye hear His words so blest:
All ye that labour, come to Me,
And I will give you rest.

*Latin Hymn.*

Jesu, the very thought of Thee
With sweetness fills my breast;
But sweeter far Thy Face to see
And in Thy presence rest!

Nor voice can sing, nor heart can frame,
Nor can the memory find,
A sweeter sound than Thy blest Name,
O Saviour of Mankind.

O Hope of every contrite heart!
O Joy of all the meek!
To those who fall, how kind Thou art!
How good to those who seek!

*S. Bernard.*

**Reading.**

When God is in the midst of a kingdom, or city, He makes it as firm as Mount Sion that cannot be removed. When He is in the midst of a soul, though calamities throng about it on all hands, and roar like the billows of the sea, yet there is a constant calm within, such a peace as the world can neither give nor take away. That is the way to be immovable in the midst of troubles, as a rock amidst the waves.—*Archbishop Leighton.*

Thou oughtest often to have recourse to the Fountain of Grace and of Divine Mercy, to the Fountain of all Goodness and all Purity, that thou mayst be healed of thy sins and passions, and be made more strong and vigilant against all the temptations and deceits of the devil.—*Thomas à Kempis.*

Perhaps nothing proves so certainly how we are related to the unseen world as our prayers. If they be tedious and irksome, cold and tasteless, it is a sure proof that our delight is not in God, and that we love Him chiefly, if not only, in the reason; that we are living if not lives of sense, at least of intellect and imagination, rather than of the will. So long as you are in this state, however much this world may lose its hold upon us, the next has not as yet won our hearts. —*Dr. Manning.*

### Death in Our Ranks.

WE regret to have to record the death of Miss Helen A. Stewart, one of the Naval Nursing Sisters at Chatham Hospital. Miss Stewart was trained at King's, and joined the naval service as a Sister at Plymouth Hospital in February, 1889. She subsequently worked for several years at the Royal Naval Hospital, Haslar (near Portsmouth), where her care and devotion to her patients was greatly appreciated. She also did good service in training the male nurses who worked in her wards—by no means an easy task. She had only been ill a few days, and died at the house of Miss Isla Stewart, her sister, matron of St. Bartholomew's Hospital in London on the night of Sunday, March 27th.

### Notes and Queries.

**The contents of the Editor's Letter-box have now reached such unwieldy proportions that it has become necessary to establish a hard and fast rule regarding Answers to Correspondents. In future, all questions requiring replies will continue to be answered in this column without any fee. If an answer is required by letter, a fee of half-a-crown must be enclosed with the note containing the enquiry. We are always pleased to help our numerous correspondents to the fullest extent, and we can trust them to sympathise in the overwhelming amount of writing which makes the new rules a necessity. Every communication must be accompanied by the writer's name and address, otherwise it will receive no attention.**

*Dental Hospitals.*

(19) Can you give me any information of dental hospitals in London, free or otherwise?—*S. R. H.*

The Dental Hospital of London, 40, Leicester Square, and the National Dental Hospital, 187-191, Great Portland Street, W.; free. There are dental departments at the London medical schools. Guy's Hospital has a large one.

*Male Nurses.*

(20) I would be very glad to know what hospitals in London take in probationers to train as male nurses, and who to write to.—*H. R.*

The National Hospital for the Paralysed and Epileptic, Queen's Square, Bloomsbury, is the only one. Apply the Matron. Asylums offer the best training available as yet to men in England.

*Why Some Probationers Pay.*

(21) How is it that probationers have to pay a premium to enter some hospitals, while at others there is a salary given? Does it make any difference in the training?—*Enquirer.*

It makes little difference now, if any. Paying probationers train only for a short time, as a rule.

*Bicycles for District Nurses.*

(22) 1. When a bicycle is provided by an association for its nurses, are they allowed to have the unlimited use of it? 2. Who is responsible for repairs and wear and tear?—*Henrietta E.*

1. Each association must make its own rules. It seems fair that the bicycle should only be used for the nurses' work, except by special permission of the lady superintendent or committee. 2. The cost of repairing and all reasonable wear and tear should be borne by the association. In fact, the rules should be much the same as those made for other conveyances the use of which is allowed to employées.

*An Opening for a Visiting Nurse.*

(23) I am a three years' certificated nurse (Guy's), and have had 14 years' experience in all sorts of nursing, medical, maternity, surgical, and massage. Frequent attacks of influenza have weakened my health, and made me feel unequal to the responsibility of a permanent post as matron. I have a certain amount of visiting work, but not enough. I am, therefore, desirous of finding an opening where there is plenty of daily work, and where I could receive a resident patient into my own home. Can you help me?—*A. F. F.*

We fear you are undertaking too much if your health is poor. The only way to obtain such work is to develop a connection where you are known.

*Dundee Royal Infirmary.*

(24) How many beds are there in Dundee Royal Infirmary? Is a three years' certificate from this hospital equal to those of the London hospitals? How can I obtain it?—*Muriel.*

There are 286 beds in Dundee Royal Infirmary. A three years' certificate from a recognised training school, such as this, will admit you to any position in the nursing world for which you are otherwise fit. Of course, the London hospitals have greater prestige, and some offer a wider experience. Still, you may earn both after obtaining your certificate. The opportunities for individual attention in the smaller schools, too, are often more valuable to a probationer than in the larger hospitals. Apply to the Matron.

*Training for Monthly Nursing.*

(25) Can you tell me of a healthy place, such as Bournemouth, where I could be trained for monthly nursing? Are the examinations difficult?—*H. B.*

It is difficult to find training schools apart from large institutions. It is quite possible for you, however, to be taught the practical work by a district midwife under a medical man, who can certify that you have attended the requisite number of cases. The examinations are easy, especially for an educated woman.

*Drugs.*

(26) Can you recommend me a book on drugs, their uses and characteristics? Please give price and publisher.—*Sister Irene.*

"Materia Medica and Therapeutics," by Dr. Mitchell Bruce, is a book largely used by medical students.

*Homes for Training Baby's Nurses.*

(27) Kindly tell me the names of homes where they train baby's nurses?—*Lilian Ashley.*

The Norland Institute for Training Children's Nurses, Notting Hill, W.; Moseley Hall Convalescent Home; Children's Nursing Home, Loughton, Essex.

### Where to Go.

A GRAND choral and orchestral concert in aid of the Westminster Hospital will be given at St. James's Hall on Wednesday, May 11th, at 8 p.m.

